# Dataset of reaching behavior for reward in social situations in mice

**DOI:** 10.1016/j.dib.2021.106773

**Published:** 2021-01-30

**Authors:** Masatoshi Ukezono, Yuji Takano

**Affiliations:** a Department of Neuropsychopharmacology, National Institute of Mental Health, National Center of Neurology and Psychiatry, Kodaira, Tokyo, Japan; b Medical Science Innovation Hub Program, RIKEN, Yokohama, Kanagawa, Japan; c Smart-Aging Research Center, Tohoku University, Sendai, Miyagi, Japan

**Keywords:** Mirror neuron system, Intention understanding, Reaching behavior, Observation

## Abstract

The data presented in this article is from a paper entitled “An experimental task to examine the mirror neuron system in mice: Laboratory mice understand the movement intentions of other mice based on their own experience” (Ukezono and Takano, 2021). This article contains individual data on reaching behavior for reward in social situations in mice. In the reaching room, the mice first learned how to acquire food by reaching their limbs. The mice that had learned reaching were placed in an observation room where they could observe the reaching activity of another mouse in the reaching room. The data includes all animals’ properties and conditions, the pairing state of another mouse (cage mate or non-cage mate), and a set of behavioral analyses. Our data have the potential to be reused for analyzing interaction behaviors of mice placed in front of rewards and developing experiments for behavioral neuroscience research on the mirror neuron system in mice.

## Specifications Table

**Table:** 

Subject	Behavioral neuroscience
Specific subject area	Mirror system Reaching behavior Observation
Type of data	Table, figure
How data were acquired	Data were collected using a reaching behavior task. The apparatus, which included a reaching room and an observation room, was made of a transparent acrylic, with a feeding table between the two sides. In the reaching room, a slit (10 mm) was created near the feeding table to allow the mice to reach for and grasp a piece of pasta, which served as a food reward. We placed two video cameras, one above and another in front of the apparatus, and recorded the animal behaviors (60 fps).
Data format	Raw and analyzed
Parameters for data collection	Data were collected from 32 C57BL/N male mice in Experiment 1 and 50 C57BL/N male mice in Experiment 2. We recorded the behavior of the mice while they observed the reaching behavior in conspecifics under different conditions, manually categorized the behavior, measured the speed by stopwatch, and measured the approach time by stopwatch.
Description of data collection	The behavioral data were collected in the same room. We placed two video cameras, one above and another in front of the apparatus, and recorded the animal behaviors (60 fps) during the learning of the reaching behavior and test session.
Data source location	Institute for Animal Experimentation Tohoku University Graduate School of Medicine. City/Town/Region: Sendai-shi, Miyagi Country: Japan
Data accessibility	Data is accessible from this article and the following data repositories. Experimental videos of Experiment 1 and Experiment 2, which were used for our manual judgment, have been uploaded to the data repository site. Repository name: Zenodo Direct URL to data of Experiment 1 in test session: https://zenodo.org/record/4286071#.X9WYstj7Q2w And Experiment 2 in test session: https://zenodo.org/record/4287815#.X9WaMNj7Q2w
Related research article	Ukezono, M., and Takano, Y. (2021). An experimental task to examine the mirror neuron system in mice: Laboratory mice understand the movement intentions of other individuals through their own experience, Behavioral Brain Research. 398, 112970. https://doi.org/10.1016/j.bbr.2020.112970

## Value of the Data

• This is a valuable dataset because mice that have learned reaching behaviors paid attention to, were in close proximity with, and observed mice demonstrating reaching behaviors of other individuals, allowing for additional analysis of these behaviors. In addition, the full data for each mouse is provided so that the reader can refer to the individual learning process.
• The data on mice paying attention to other individuals and observational learning in mice will be useful to psychologists in the learning and social fields. It is also a useful dataset for behavioral neuroscientists as it can be used to record neural activity during this experimental task.
• As it is possible to analyze how reaching behavior changes depending on whether the mouse has learned the behavior or not, this dataset can provide insight into understanding intentions and help propagate research in this field, including research on the mirror neuron system.

## 1 Data Description

The data presented in this article is from a paper entitled “An experimental task to examine the mirror neuron system in mice: Laboratory mice understand the movement intentions of other mice based on their own experience” (Ukezono and Takano, 2021). This article contains data on reaching behavior for reward in social situations of individual mice. In addition, there is information on training time and data on the follow-up elements of Experiment 1 and Experiment 2, and these data were not provided in the previous paper [1].

In Experiment 1, we assessed whether a mouse observed the reaching behavior of other individuals after it learned to reach for food. As a control condition, we assessed the behavior of mice that had not learned to reach and determined whether they observed the reaching behavior of other individuals.

**Fig. 1:**
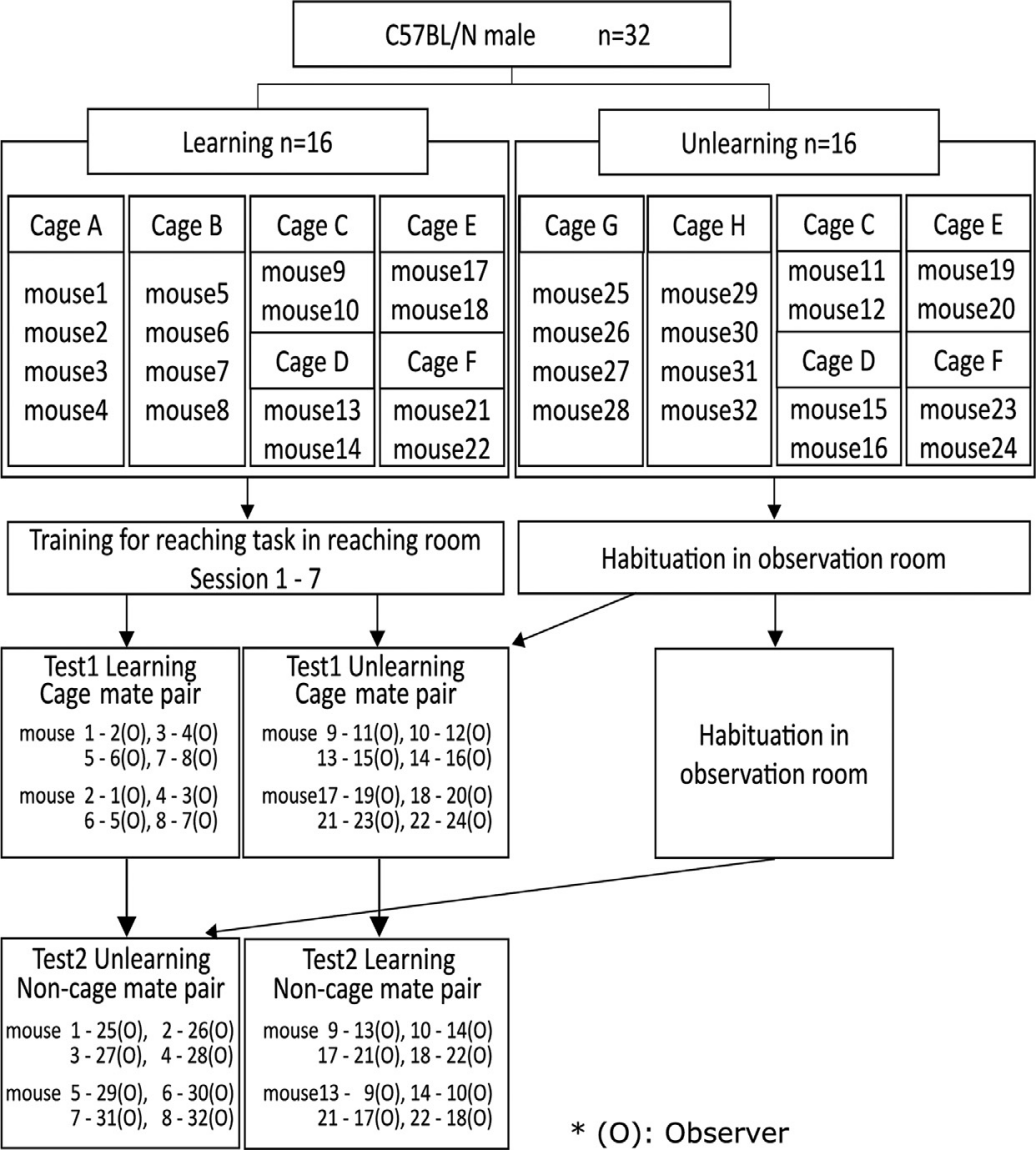
Flow-chart of Experiment 1. To distribute cage mate and non-cage mate pairings, half were assigned within one cage of the learning and unlearned groups. The mice of the Learning group were trained in reaching behavior twice daily in the reaching room. For the Unlearning group, the mice were put in the observation room for 10 min while keeping the learning room empty during each session. Following session 7 of Experiment 1, test 1, an observation test, was conducted on the same day. Thereafter, test 2 was conducted the next day. *(O) indicates observer role in the test session.

Fig. 1 shows the characteristics of each mouse and the experimental schedule, and Table 1 shows the detailed characteristics of all 32 mice in Experiment 1. After finishing training, behavioral tests were conducted for pairs, and the combinations were cage mate or non-cage mates. In the test, we had the learned reaching group (Learning group) and the unlearned group (Unlearning group) observe another individual's reaching behavior for food.

**Table 1:** The characteristics of all mice in Experiment 1.

ID	Condition	Session 1	Session 2	Session 3	Session 4	Session 5	Session 6	Session 7	Test
ex2_mouse1	Learning	1200	1200	1200	1200	1109	768	555	401
ex2_mouse2	Learning	1200	1200	1200	1200	898	560	733	650
ex2_mouse3	Unlearning	1200	1200	1200	1200	1091	1057	1191	603
ex2_mouse4	Unlearning	1200	1200	1200	1200	1200	1200	755	603
ex2_mouse5	Empty	1200	1200	1200	1200	1042	571	534	603
ex2_mouse6	Empty	1200	1200	1200	1200	815	770	502	603
ex2_mouse11	Learning	1200	1200	1200	1200	1200	1037	907	605
ex2_mouse12	Learning	1200	1200	1200	1200	784	760	578	697
ex2_mouse13	Unlearning	1200	1200	1200	1200	1200	1181	1198	603
ex2_mouse14	Unlearning	1200	1200	1200	1200	976	788	1199	603
ex2_mouse15	Empty	1200	1200	1200	738	651	573	381	603
ex2_mouse16	Empty	1200	1200	1200	1200	1159	908	843	603
ex2_mouse21	Learning	1200	1200	1200	933	599	622	640	519
ex2_mouse22	Learning	1200	1200	1200	1200	1200	838	776	524
ex2_mouse23	Unlearning	1200	1200	1200	1011	774	424	460	603
ex2_mouse24	Unlearning	1200	1200	1200	974	711	736	617	603
ex2_mouse25	Empty	1200	1200	802	1200	497	534	412	603
ex2_mouse26	Empty	1200	1200	1200	895	429	376	478	603
ex2_mouse31	Learning	1200	1200	1200	1200	808	890	754	536
ex2_mouse32	Learning	1200	1200	1200	1200	740	801	715	557
ex2_mouse33	Unlearning	1200	1200	1200	1200	1041	775	619	603
ex2_mouse34	Unlearning	1200	1200	1200	1200	1013	595	1195	603
ex2_mouse35	Empty	1200	1200	1200	1200	750	487	389	603
ex2_mouse36	Empty	1200	1200	1200	1200	1200	851	581	603
ex2_mouse41	Learning	1200	1200	1200	1200	600	1200	964	543
ex2_mouse42	Learning	1200	1200	1200	1200	753	1014	531	553
ex2_mouse43	Unlearning	1200	1200	1200	1200	1200	637	483	603
ex2_mouse44	Unlearning	1200	1200	1200	1200	1200	1200	628	603
ex2_mouse45	Empty	1200	1200	1200	1200	1200	671	481	603
ex2_mouse46	Empty	1200	1200	1200	1200	1200	1043	748	603
ex2_mouse7	demonstrator	1200	1200	1200	1200	1099	1200	393	401
ex2_mouse8	demonstrator	1200	1200	1200	1200	1200	854	845	650
ex2_mouse9	demonstrator	600	600	600	600	600	600	600	603
ex2_mouse10	demonstrator	600	600	600	600	600	600	600	603
ex2_mouse17	demonstrator	1200	1200	1039	925	625	843	562	605
ex2_mouse18	demonstrator	1200	1200	802	920	730	648	1079	697
ex2_mouse19	demonstrator	600	600	600	600	600	600	600	603
ex2_mouse20	demonstrator	600	600	600	600	600	600	600	603
ex2_mouse27	demonstrator	1200	1200	1167	1035	637	505	347	519
ex2_mouse28	demonstrator	1200	1200	1200	1200	696	484	650	524
ex2_mouse29	demonstrator	600	600	600	600	600	600	600	603
ex2_mouse30	demonstrator	600	600	600	600	600	600	600	603
ex2_mouse37	demonstrator	1200	1200	1200	1200	1041	715	683	536
ex2_mouse38	demonstrator	1200	1200	1200	1200	1200	1200	669	557
ex2_mouse39	demonstrator	600	600	600	600	600	600	600	603
ex2_mouse40	demonstrator	600	600	600	600	600	600	600	603
ex2_mouse47	demonstrator	1200	1200	1200	1200	1200	118	418	543
ex2_mouse48	demonstrator	1200	1200	1200	1200	1150	862	532	553
ex2_mouse49	demonstrator	600	600	600	600	600	600	600	603
ex2_mouse50	demonstrator	600	600	600	600	600	600	600	603

Table 2 shows the success rate of reaching for each mouse during training sessions 3 to 7 in Experiment 1. The reason for omitting sessions 1 and 2 is that during these sessions the mice were only trained to reach and grasp for the pasta with their forepaws. The success rate was calculated by dividing the number of successful reaching attempts by the total number of trials in a session, which was 20. Successful reaching was defined as reaching for and grasping a piece of pasta and eating it.

**Table 2:** Success rate of reaching in the training sessions in Experiment 1.

ID	Session 3	Session 4	Session 5	Session 6	Session 7
mouse1	0%	0%	25%	35%	75%
mouse2	5%	30%	20%	55%	70%
mouse3	15%	45%	45%	45%	90%
mouse4	40%	35%	45%	60%	70%
mouse5	25%	30%	50%	75%	65%
mouse6	65%	50%	45%	90%	80%
mouse7	45%	15%	40%	85%	75%
mouse8	20%	20%	35%	45%	70%
mouse9	65%	70%	80%	70%	75%
mouse10	60%	60%	90%	75%	70%
mouse11	50%	80%	75%	70%	75%
mouse12	75%	95%	80%	80%	90%
mouse13	70%	75%	90%	85%	75%
mouse14	65%	75%	85%	80%	100%
mouse15	75%	65%	80%	90%	75%
mouse16	80%	80%	90%	90%	80%

To eliminate behaviors other than reaching, the mice were trained to turn around on the spot before reaching. Table 3 shows the spinning time in session 7 (no observer), unlearning observer in the test session, and learning observer in the test session. Previous studies have used an analysis of the difference in spinning time to indicate the effects by the presence or absence of social perception between two individuals [1], [2].

**Table 3:** Speed of spinning in Experiment 1.

ID	Session 7	Unlearning	Learning
mouse1	1.86	1.30	1.12
mouse2	1.49	1.31	1.25
mouse3	1.73	1.53	1.45
mouse4	1.26	1.46	1.20
mouse5	1.21	1.23	1.03
mouse6	1.26	1.23	0.92
mouse7	1.36	1.23	1.04
mouse8	1.94	1.26	1.16
mouse9	1.23	1.22	0.85
mouse10	1.47	1.21	0.87
mouse11	1.54	1.49	1.29
mouse12	1.44	1.26	0.92
mouse13	1.72	1.60	1.07
mouse14	1.59	1.30	1.28
mouse15	1.37	1.17	1.04
mouse16	1.42	1.16	0.92

**Fig. 2:**
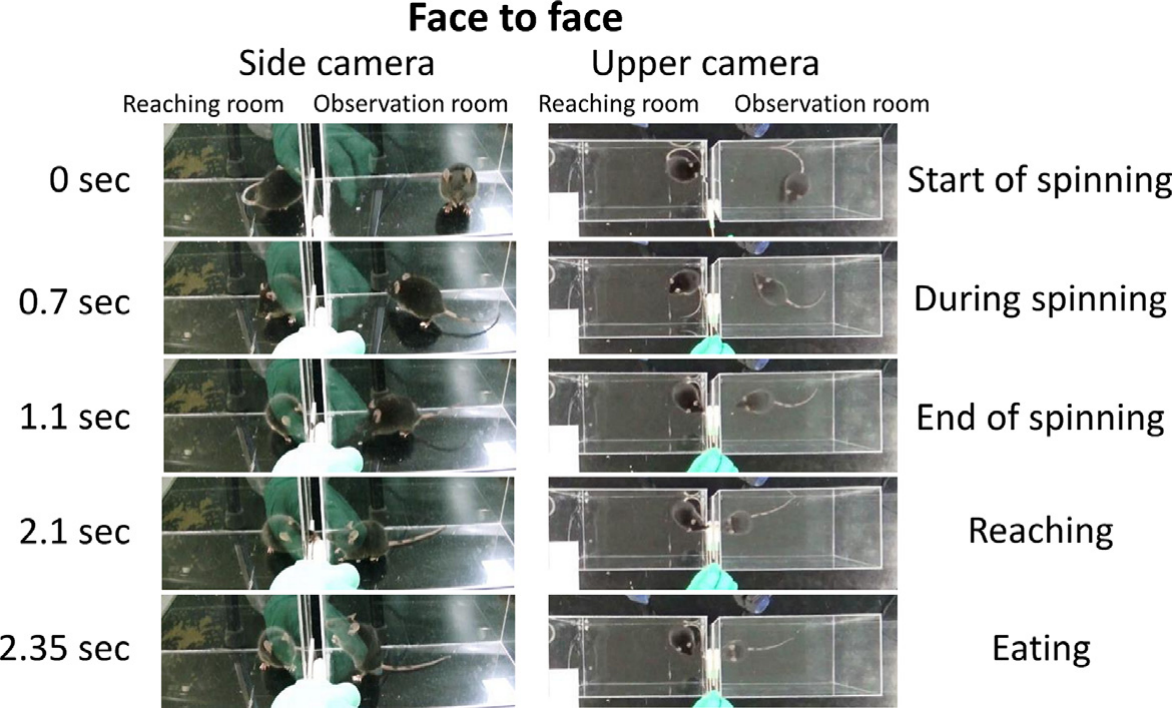
Example of “face to face” behavior observed using the side and upper camera. The first picture indicates the starting of spinning and is set to 0 sec. Reaching behavior was performed after the end of the spin, and at the moment when the mouse touched the pasta, the observer's behavior was classified into the following three categories: “face to face,” “ambiguous,” or “not paying attention.” The “face to face” behavior is defined as the two heads being in a straight line at the time of reaching behavior.

**Fig. 3:**
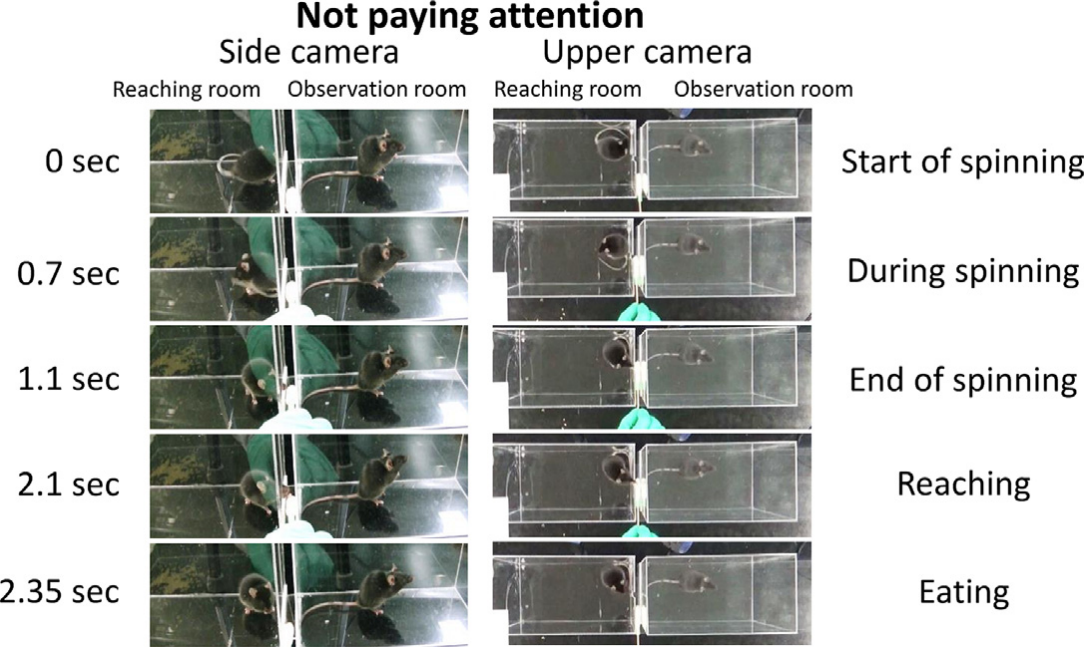
Example of the “not paying attention” behavior as observed from the side and upper cameras. “Not paying attention” behavior was defined as the observer looking away, beyond 90°, from the slit.

Fig. 2, Fig. 3 show the examples of “face to face” and “not paying attention” behavior of the observer mouse in the test session in Experiment 1. From the data in the video, we manually grouped the observer's behavior into three categories: “face to face,” “ambiguous,” and “not paying attention.” The videos on which these ratings are based on are accessible from the sites noted in the Data accessibility section. “Face to face’’ was noted when the two heads were in a straight line in videos captured by the side and upper cameras. “Not paying attention” was noted when the observer was looking away, beyond 90°, from the partner that was reaching for a pasta independent of position. “Ambiguous” was noted when the situation did not fit the definition of “face to face” or “not paying attention.”

Table 4 (Learning group) and Table 5 (Unlearning group) are the coded data of the observing individuals’ behavior when the reaching individual was reaching during the test session. While the other mouse was reaching, we categorized the observer's behavior as “face to face,” “ambiguous,” or “not paying attention” and counted the number of times they presented with each of these behaviors. The occurrence ratio was calculated by dividing the number of times each behavioral category was observed by the total number of trials, which was 20.

**Table 4:** Frequency of each behavioral category for mice in the Learning group in Experiment 1.

ID	Condtion	F to F	Ambiguous	Not paying attention
mouse1	Learning	0%	50%	50%
mouse2	Learning	35%	35%	30%
mouse3	Learning	65%	15%	20%
mouse4	Learning	30%	20%	50%
mouse5	Learning	0%	55%	45%
mouse6	Learning	25%	20%	55%
mouse7	Learning	30%	20%	50%
mouse8	Learning	25%	25%	50%
mouse9	Learning	30%	25%	45%
mouse10	Learning	15%	45%	40%
mouse11	Learning	25%	35%	40%
mouse12	Learning	35%	25%	40%
mouse13	Learning	25%	30%	45%
mouse14	Learning	30%	30%	40%
mouse15	Learning	10%	40%	50%
mouse16	Learning	20%	25%	55%

**Table 5:** Frequency of each behavioral category for mice in the Unlearning group in Experiment 1.

ID	Condtion	F to F	Ambiguous	Not paying attention
mouse17	Unlearning	0%	15%	85%
mouse18	Unlearning	5%	20%	75%
mouse19	Unlearning	5%	15%	80%
mouse20	Unlearning	10%	25%	65%
mouse21	Unlearning	5%	40%	55%
mouse22	Unlearning	10%	10%	80%
mouse23	Unlearning	5%	30%	65%
mouse24	Unlearning	5%	40%	55%
mouse25	Unlearning	5%	30%	65%
mouse26	Unlearning	20%	45%	35%
mouse27	Unlearning	15%	40%	45%
mouse28	Unlearning	10%	25%	65%
mouse29	Unlearning	15%	40%	45%
mouse30	Unlearning	5%	40%	55%
mouse31	Unlearning	10%	50%	40%
mouse32	Unlearning	10%	30%	60%

**Fig. 4:**
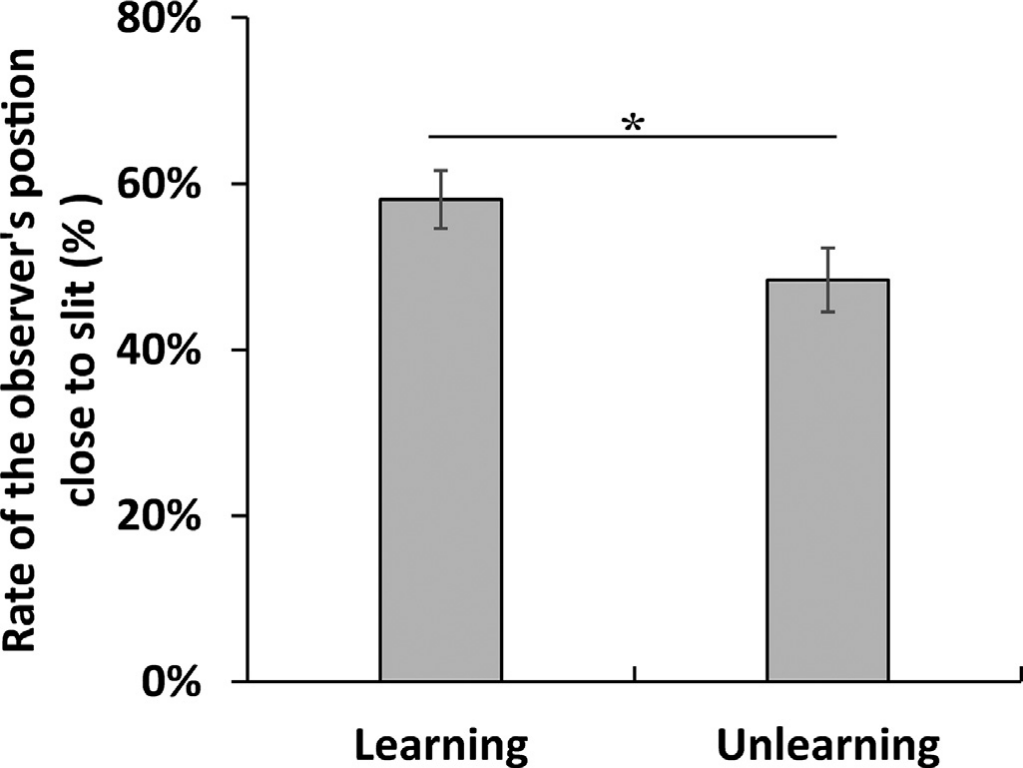
The occurrence rate of the observer's position being close to the slit in the observation room during the test session in Experiment 1. The rate of the observer's position being close to the slit was significantly higher in the Learning group than that in the Unlearning group. Error bars represent the standard error of the mean. *p < 0.05.

Table 6 (Individual data) and Fig. 4 (Averaging data) show the amount of time mice spent near the slit in the observation room during the test session in Experiment 1. When the animal was on the slit side in the middle of the observation box, we defined it as being close to the slit. The amount of time the observer's position was close to the slit, as seen from the upper camera, was determined. The frequency of near position by observer was calculated by dividing the time close to the slit by the total time of the experiment for the Learning group (mean = 58.1%, standard deviation [SD] = 10.69) and Unlearning group (mean = 48.4%, SD = 11.83). We compared the rate of observer position between the Learning and Unlearning groups. This comparison was significant (*t*(30) = 2.35, *p* < 0.05).

**Table 6:** Occurrence rate of the observer's position being close to the slit in Experiment 1.

ID	Condition	Rate of the observer's position close to slit
mouse1	Learning	55.44%
mouse2	Learning	41.22%
mouse3	Learning	68.34%
mouse4	Learning	62.83%
mouse5	Learning	69.96%
mouse6	Learning	45.32%
mouse7	Learning	77.50%
mouse8	Learning	56.90%
mouse9	Learning	59.21%
mouse10	Learning	40.04%
mouse11	Learning	48.48%
mouse12	Learning	63.22%
mouse13	Learning	68.45%
mouse14	Learning	68.20%
mouse15	Learning	51.65%
mouse16	Learning	52.72%
mouse17	Unlearning	36.51%
mouse18	Unlearning	61.75%
mouse19	Unlearning	23.86%
mouse20	Unlearning	37.88%
mouse21	Unlearning	41.42%
mouse22	Unlearning	45.63%
mouse23	Unlearning	31.77%
mouse24	Unlearning	47.21%
mouse25	Unlearning	54.27%
mouse26	Unlearning	59.17%
mouse27	Unlearning	55.16%
mouse28	Unlearning	42.37%
mouse29	Unlearning	52.76%
mouse30	Unlearning	65.52%
mouse31	Unlearning	53.32%
mouse32	Unlearning	65.84%

**Fig. 5:**
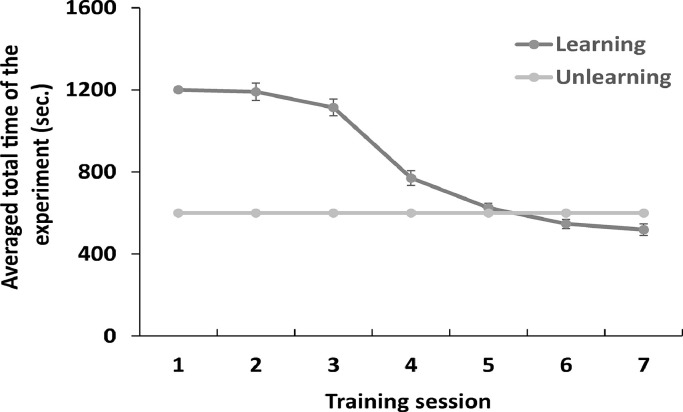
The total experiment time in the training session in Experiment 1. In the Learning group, the mice were trained in reaching behavior via 20 trials in a session twice a day in the reaching room. The time limit of each session was 20 min. In the Unlearning group, the mice were put in the observation room for 10 min without pasta while keeping the reaching room empty during each session. Error bars represent standard error of the mean.

**Fig. 6:**
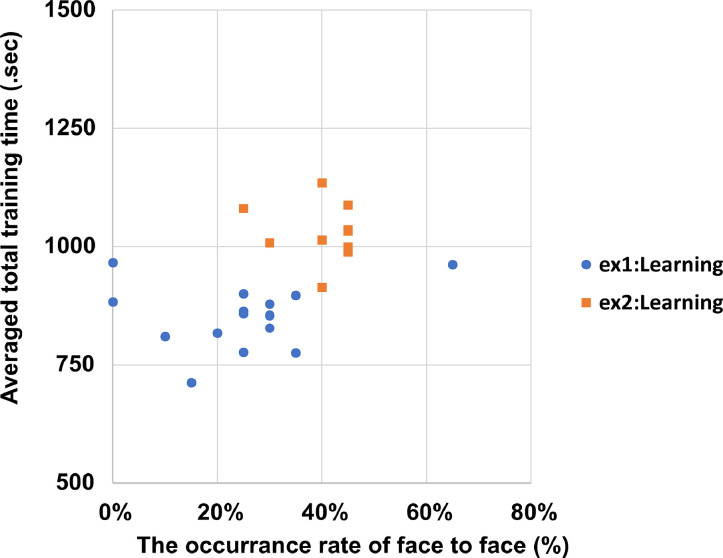
The relationship between training time and occurrence of “face to face” behavior. The occurrence rate was calculated by dividing the number of times “face to face” behavior was noted by the total number of trials, which was 20. The average of total training time was calculated from session 1 to session 7 for each mouse. Ex1: Experiment 1, Ex2: Experiment 2, learning: learning observer.

Table 7 (Individual data) and Fig. 5 (Averaging data) show the total experimental time in Experiment 1. The total training time for each individual in training sessions 1–7 and the average total training time were calculated to determine if there was a difference in training time between the Learning and Unlearning groups. The total training time between the Learning and Unlearning groups was significantly different (*t*(15) = 15.23, *p* < 0.01). The time spent in the apparatus was longer for the Learning group. Fig. 6 shows the correlation between the average total training time and the frequency of “face to face” behavior. There was no correlation between training time and the occurrence of “face to face” behavior in the test sessions of Experiments 1 and 2 (Ex1: *r* = -0.18, *p* = 0.52, *n.s.*; Ex2: *r* = -0.13, *p* = 0.72, *n.s.*).

**Table 7:** Total time duration of learning sessions and test sessions. The Unlearning group spent 600 s in each training session, from session 1 to session 7.

	Total time of experiment (s)								
ID	Session 1	Session 2	Session 3	Session 4	Session 5	Session 6	Session 7	Test 1	Test 2
mouse1	1200	1200	1200	923	788	881	572	558	578
mouse2	1200	1200	1200	856	659	671	491	495	502
mouse3	1200	1200	1063	963	813	788	704	696	704
mouse4	1200	1200	1032	700	672	558	623	605	623
mouse5	1200	1200	1023	922	572	608	656	608	618
mouse6	1200	1200	1124	820	710	553	693	553	607
mouse7	1200	1200	1155	818	643	569	563	569	598
mouse8	1200	1200	1032	883	667	536	527	536	538
mouse9	1200	1200	1200	877	574	382	362	433	412
mouse10	1200	1200	911	498	464	373	341	409	412
mouse11	1200	1200	1058	834	627	568	520	640	515
mouse12	1200	1200	1200	552	592	334	350	465	459
mouse13	1200	1050	1200	599	378	533	476	453	429
mouse14	1200	1200	1141	803	671	487	473	493	537
mouse15	1200	1200	1132	678	586	448	425	437	427
mouse16	1200	1200	1155	596	588	465	517	486	416

In Experiment 2, after training the mice to perform reaching behavior as done in Experiment 1, data on searching for other individuals performing reaching behavior, searching for untrained individuals, and searching for empty boxes were collected.

Table 8 provides the characteristics of the observer mice and their assigned conditions (a condition in which the demonstrator to be observed has learned or not learned reaching behavior or a condition in which an empty box is present) in the test session of Experiment 2. Table 9 shows the characteristics of the mice (Learning or Unlearning group) used as demonstrators in the test session.

**Table 8:** The characteristics of mice used as observers in Experiment 2.

ID	Week age	Weight at the start (g)	Cage	Learning or Unlearning	Condition	Test		Weight at the end (g)
						partner		
ex2_mouse1	7	19	Cage1	Learning	Learning	mouse7	Non-cage mate	19.6
ex2_mouse2	7	18.3	Cage1	Learning	Learning	mouse8	Non-cage mate	18.8
ex2_mouse3	7	18.4	Cage1	Learning	Unlearning	mouse9	Non-cage mate	18.8
ex2_mouse4	7	18.4	Cage1	Learning	Unlearning	mouse10	Non-cage mate	18.4
ex2_mouse5	7	17.5	Cage2	Learning	Empty			18.5
ex2_mouse6	7	20.2	Cage2	Learning	Empty			20.2
ex2_mouse11	6	19.3	Cage4	Learning	Learning	mouse17	Non-cage mate	19.6
ex2_mouse12	6	19.2	Cage4	Learning	Learning	mouse18	Non-cage mate	19.3
ex2_mouse13	6	18.6	Cage4	Learning	Unlearning	mouse19	Non-cage mate	18.7
ex2_mouse14	6	19.3	Cage4	Learning	Unlearning	mouse20	Non-cage mate	19.1
ex2_mouse15	6	19.1	Cage5	Learning	Empty			18.5
ex2_mouse16	6	18.7	Cage5	Learning	Empty			18.8
ex2_mouse21	5	21.2	Cage7	Learning	Learning	mouse27	Non-cage mate	19.8
ex2_mouse22	5	20.1	Cage7	Learning	Learning	mouse28	Non-cage mate	20.4
ex2_mouse23	5	20.9	Cage7	Learning	Unlearning	mouse29	Non-cage mate	20.1
ex2_mouse24	5	19.9	Cage7	Learning	Unlearning	mouse30	Non-cage mate	18.9
ex2_mouse25	5	21.5	Cage8	Learning	Empty			20.7
ex2_mouse26	5	20.8	Cage8	Learning	Empty			20.1
ex2_mouse31	6	20.6	Cage10	Learning	Learning	mouse37	Non-cage mate	19.6
ex2_mouse32	6	20.7	Cage10	Learning	Learning	mouse38	Non-cage mate	19.9
ex2_mouse33	6	21.6	Cage10	Learning	Unlearning	mouse39	Non-cage mate	20.9
ex2_mouse34	6	22.3	Cage10	Learning	Unlearning	mouse40	Non-cage mate	20.7
ex2_mouse35	6	20.2	Cage11	Learning	Empty			19.9
ex2_mouse36	6	20.3	Cage11	Learning	Empty			19.5
ex2_mouse41	6	18.8	Cage13	Learning	Learning	mouse47	Non-cage mate	18.7
ex2_mouse42	6	17.8	Cage13	Learning	Learning	mouse48	Non-cage mate	18.3
ex2_mouse43	6	21	Cage13	Learning	Unlearning	mouse49	Non-cage mate	19.4
ex2_mouse44	6	19.3	Cage13	Learning	Unlearning	mouse50	Non-cage mate	18.4
ex2_mouse45	6	20.6	Cage14	Learning	Empty			19.3
ex2_mouse46	6	19.9	Cage14	Learning	Empty			18.7

**Table 9:** The characteristics of mice used as demonstrators in the test session in Experiment 2.

ID	Week age	Weight at the start (g)	Cage	Learning or Unlearning	Test partner		Weight at the end (g)
ex2_mouse7	7	21.3	Cage2	Learning	mouse1	Non-cage mate	21.8
ex2_mouse8	7	19	Cage2	Learning	mouse2	Non-cage mate	19.5
ex2_mouse9	7	18.5	Cage3	Unlearning	mouse3	Non-cage mate	19.1
ex2_mouse10	7	17.8	Cage3	Unlearning	mouse4	Non-cage mate	18.4
ex2_mouse17	6	20.1	Cage5	Learning	mouse11	Non-cage mate	19.8
ex2_mouse18	6	19.2	Cage5	Learning	mouse12	Non-cage mate	18.9
ex2_mouse19	6	18.9	Cage6	Unlearning	mouse13	Non-cage mate	18.5
ex2_mouse20	6	18.9	Cage6	Unlearning	mouse14	Non-cage mate	18.7
ex2_mouse27	5	19.6	Cage8	Learning	mouse21	Non-cage mate	19.4
ex2_mouse28	5	18.4	Cage8	Learning	mouse22	Non-cage mate	17.3
ex2_mouse29	5	18.3	Cage9	Unlearning	mouse23	Non-cage mate	18
ex2_mouse30	5	17.4	Cage9	Unlearning	mouse24	Non-cage mate	17
ex2_mouse37	6	19.8	Cage11	Learning	mouse31	Non-cage mate	18.9
ex2_mouse38	6	22.1	Cage11	Learning	mouse32	Non-cage mate	21.1
ex2_mouse39	6	22.2	Cage12	Unlearning	mouse33	Non-cage mate	21.2
ex2_mouse40	6	22.1	Cage12	Unlearning	mouse34	Non-cage mate	20.7
ex2_mouse47	6	19.5	Cage14	Learning	mouse41	Non-cage mate	18.3
ex2_mouse48	6	20.9	Cage14	Learning	mouse42	Non-cage mate	19.3
ex2_mouse49	6	20.9	Cage15	Unlearning	mouse43	Non-cage mate	18.8
ex2_mouse50	6	23.4	Cage15	Unlearning	mouse44	Non-cage mate	21.8

Table 10 shows the observer's position information, which was calculated in the same way as that used for the information in Table 6. Table 11 shows the success rate of reaching for each mouse in the training sessions 3 to 7 in Experiment 2; it is similar to Table 2 for Experiment 1. Table 12 shows the spinning time in session 7 and in the test session with only Learning-Learning pairs. Table 13 shows the behavior of the observer at the time when the other mouse was reaching during the test session in Experiment 2, similar to that shown in Tables 4 and 5. Table 14 shows the total experimental time in Experiment 2, similar to that in Table 7. These data (Table 11, Table 12, Table 13, Table 14) allow us to confirm whether the mice learned reaching equally in both Experiment 1 and Experiment 2.

**Table 10:** The occurrence rate of the observer's position being close to the slit in Experiment 2.

ID	Condition	Rate of the observer's position close to slit
ex2_mouse1	Learning	64.09%
ex2_mouse2	Learning	49.69%
ex2_mouse3	Unlearning	39.47%
ex2_mouse4	Unlearning	59.20%
ex2_mouse5	Empty	51.41%
ex2_mouse6	Empty	51.74%
ex2_mouse11	Learning	58.68%
ex2_mouse12	Learning	78.91%
ex2_mouse13	Unlearning	53.07%
ex2_mouse14	Unlearning	38.31%
ex2_mouse15	Empty	51.08%
ex2_mouse16	Empty	51.58%
ex2_mouse21	Learning	73.41%
ex2_mouse22	Learning	63.55%
ex2_mouse23	Unlearning	46.10%
ex2_mouse24	Unlearning	52.24%
ex2_mouse25	Empty	51.58%
ex2_mouse26	Empty	44.44%
ex2_mouse31	Learning	69.22%
ex2_mouse32	Learning	55.12%
ex2_mouse33	Unlearning	52.07%
ex2_mouse34	Unlearning	47.10%
ex2_mouse35	Empty	48.26%
ex2_mouse36	Empty	42.79%
ex2_mouse41	Learning	53.96%
ex2_mouse42	Learning	69.44%
ex2_mouse43	Unlearning	56.88%
ex2_mouse44	Unlearning	57.05%
ex2_mouse45	Empty	53.90%
ex2_mouse46	Empty	43.62%

**Table 11:** Success rate of reaching in the training sessions in Experiment 2.

ID	Session 3	Session 4	Session 5	Session 6	Session 7
ex2_mouse1	0%	0%	35%	80%	70%
ex2_mouse2	0%	0%	20%	55%	75%
ex2_mouse3	0%	5%	25%	45%	65%
ex2_mouse4	0%	0%	5%	50%	70%
ex2_mouse5	5%	20%	50%	80%	80%
ex2_mouse6	0%	50%	45%	15%	70%
ex2_mouse7	0%	25%	50%	50%	80%
ex2_mouse8	0%	0%	5%	50%	70%
ex2_mouse11	0%	0%	60%	40%	65%
ex2_mouse12	0%	0%	50%	65%	70%
ex2_mouse13	0%	15%	30%	30%	65%
ex2_mouse14	30%	15%	45%	55%	75%
ex2_mouse15	35%	55%	65%	75%	70%
ex2_mouse16	30%	40%	35%	35%	65%
ex2_mouse17	60%	65%	50%	50%	75%
ex2_mouse18	40%	40%	55%	45%	65%
ex2_mouse21	25%	65%	15%	55%	65%
ex2_mouse22	25%	35%	65%	65%	70%
ex2_mouse23	50%	70%	85%	50%	70%
ex2_mouse24	40%	45%	50%	45%	70%
ex2_mouse25	40%	80%	80%	80%	80%
ex2_mouse26	30%	75%	50%	40%	80%
ex2_mouse27	40%	50%	55%	55%	70%
ex2_mouse28	45%	55%	55%	70%	75%
ex2_mouse31	0%	50%	35%	25%	65%
ex2_mouse32	0%	70%	75%	60%	80%
ex2_mouse33	15%	35%	60%	40%	65%
ex2_mouse34	0%	35%	55%	50%	65%
ex2_mouse35	0%	30%	60%	70%	70%
ex2_mouse36	20%	55%	70%	70%	65%
ex2_mouse37	0%	0%	80%	40%	65%
ex2_mouse38	0%	5%	55%	80%	80%
ex2_mouse41	15%	50%	15%	20%	70%
ex2_mouse42	15%	65%	35%	65%	85%
ex2_mouse43	35%	35%	55%	65%	65%
ex2_mouse44	0%	35%	35%	30%	65%
ex2_mouse45	25%	40%	60%	50%	70%
ex2_mouse46	10%	30%	45%	90%	75%
ex2_mouse47	0%	30%	5%	80%	75%
ex2_mouse48	10%	30%	45%	65%	75%

**Table 12:** Speed of spinning in Experiment 2 (in the test session, only learning-learning pair).

ID	Condition	Session 7	Test
ex2_mouse1	Learning	1.95	
ex2_mouse2	Learning	1.94	
ex2_mouse3	Unlearning	2.58	
ex2_mouse4	Unlearning	1.99	
ex2_mouse5	Empty	1.25	
ex2_mouse6	Empty	2.04	
ex2_mouse7		2.03	1.11
ex2_mouse8		2.51	1.14
ex2_mouse11	Learning	1.69	
ex2_mouse12	Learning	1.69	
ex2_mouse13	Unlearning	2.18	
ex2_mouse14	Unlearning	1.83	
ex2_mouse15	Empty	1.47	
ex2_mouse16	Empty	1.26	
ex2_mouse17		1.74	1.31
ex2_mouse18		1.70	1.42
ex2_mouse21	Learning	1.97	
ex2_mouse22	Learning	1.56	
ex2_mouse23	Unlearning	1.25	
ex2_mouse24	Unlearning	1.28	
ex2_mouse25	Empty	2.02	
ex2_mouse26	Empty	1.46	
ex2_mouse27		1.85	1.05
ex2_mouse28		1.36	1.02
ex2_mouse31	Learning	1.63	
ex2_mouse32	Learning	1.54	
ex2_mouse33	Unlearning	1.32	
ex2_mouse34	Unlearning	1.99	
ex2_mouse35	Empty	1.35	
ex2_mouse36	Empty	1.20	
ex2_mouse37		1.51	1.21
ex2_mouse38		1.33	1.03
ex2_mouse41	Learning	1.45	
ex2_mouse42	Learning	1.31	
ex2_mouse43	Unlearning	1.34	
ex2_mouse44	Unlearning	1.20	
ex2_mouse45	Empty	1.31	
ex2_mouse46	Empty	1.37	
ex2_mouse47		1.20	1.09
ex2_mouse48		1.32	1.28

**Table 13:** Frequency of behavioral categories of learning-learning pair in Experiment 2.

ID	F to F	Ambiguous	Not paying attention
ex2_mouse1	45%	30%	25%
ex2_mouse2	45%	25%	30%
ex2_mouse11	40%	25%	35%
ex2_mouse12	45%	30%	25%
ex2_mouse21	40%	25%	35%
ex2_mouse22	45%	30%	25%
ex2_mouse31	45%	30%	25%
ex2_mouse32	30%	35%	35%
ex2_mouse41	25%	35%	40%
ex2_mouse42	40%	35%	25%

**Table 14:** Total time duration of learning sessions and test session in Experiment 2.

ID	Condition	Session 1	Session 2	Session 3	Session 4	Session 5	Session 6	Session 7	Test
ex2_mouse1	Learning	1200	1200	1200	1200	1109	768	555	401
ex2_mouse2	Learning	1200	1200	1200	1200	898	560	733	650
ex2_mouse3	Unlearning	1200	1200	1200	1200	1091	1057	1191	603
ex2_mouse4	Unlearning	1200	1200	1200	1200	1200	1200	755	603
ex2_mouse5	Empty	1200	1200	1200	1200	1042	571	534	603
ex2_mouse6	Empty	1200	1200	1200	1200	815	770	502	603
ex2_mouse11	Learning	1200	1200	1200	1200	1200	1037	907	605
ex2_mouse12	Learning	1200	1200	1200	1200	784	760	578	697
ex2_mouse13	Unlearning	1200	1200	1200	1200	1200	1181	1198	603
ex2_mouse14	Unlearning	1200	1200	1200	1200	976	788	1199	603
ex2_mouse15	Empty	1200	1200	1200	738	651	573	381	603
ex2_mouse16	Empty	1200	1200	1200	1200	1159	908	843	603
ex2_mouse21	Learning	1200	1200	1200	933	599	622	640	519
ex2_mouse22	Learning	1200	1200	1200	1200	1200	838	776	524
ex2_mouse23	Unlearning	1200	1200	1200	1011	774	424	460	603
ex2_mouse24	Unlearning	1200	1200	1200	974	711	736	617	603
ex2_mouse25	Empty	1200	1200	802	1200	497	534	412	603
ex2_mouse26	Empty	1200	1200	1200	895	429	376	478	603
ex2_mouse31	Learning	1200	1200	1200	1200	808	890	754	536
ex2_mouse32	Learning	1200	1200	1200	1200	740	801	715	557
ex2_mouse33	Unlearning	1200	1200	1200	1200	1041	775	619	603
ex2_mouse34	Unlearning	1200	1200	1200	1200	1013	595	1195	603
ex2_mouse35	Empty	1200	1200	1200	1200	750	487	389	603
ex2_mouse36	Empty	1200	1200	1200	1200	1200	851	581	603
ex2_mouse41	Learning	1200	1200	1200	1200	600	1200	964	543
ex2_mouse42	Learning	1200	1200	1200	1200	753	1014	531	553
ex2_mouse43	Unlearning	1200	1200	1200	1200	1200	637	483	603
ex2_mouse44	Unlearning	1200	1200	1200	1200	1200	1200	628	603
ex2_mouse45	Empty	1200	1200	1200	1200	1200	671	481	603
ex2_mouse46	Empty	1200	1200	1200	1200	1200	1043	748	603
ex2_mouse7	Demonstrator	1200	1200	1200	1200	1099	1200	393	401
ex2_mouse8	Demonstrator	1200	1200	1200	1200	1200	854	845	650
ex2_mouse9	Demonstrator	600	600	600	600	600	600	600	603
ex2_mouse10	Demonstrator	600	600	600	600	600	600	600	603
ex2_mouse17	Demonstrator	1200	1200	1039	925	625	843	562	605
ex2_mouse18	Demonstrator	1200	1200	802	920	730	648	1079	697
ex2_mouse19	Demonstrator	600	600	600	600	600	600	600	603
ex2_mouse20	Demonstrator	600	600	600	600	600	600	600	603
ex2_mouse27	Demonstrator	1200	1200	1167	1035	637	505	347	519
ex2_mouse28	Demonstrator	1200	1200	1200	1200	696	484	650	524
ex2_mouse29	Demonstrator	600	600	600	600	600	600	600	603
ex2_mouse30	Demonstrator	600	600	600	600	600	600	600	603
ex2_mouse37	Demonstrator	1200	1200	1200	1200	1041	715	683	536
ex2_mouse38	Demonstrator	1200	1200	1200	1200	1200	1200	669	557
ex2_mouse39	Demonstrator	600	600	600	600	600	600	600	603
ex2_mouse40	Demonstrator	600	600	600	600	600	600	600	603
ex2_mouse47	Demonstrator	1200	1200	1200	1200	1200	118	418	543
ex2_mouse48	Demonstrator	1200	1200	1200	1200	1150	862	532	553
ex2_mouse49	Demonstrator	600	600	600	600	600	600	600	603
ex2_mouse50	Demonstrator	600	600	600	600	600	600	600	603

The raw data for Fig. 4-6 and Table 2, Table 3, Table 4, Table 5, Table 6, Table 7, Table 10, Table 11, Table 12, Table 13, Table 14 are created from the “RawData.xlsx” of the supplemental material. The experimental videos that are the source of the manually coded data (Fig. 2-3) have been uploaded to the Zenodo repository.

## 2 Experimental Design, Materials, and Methods

### 2.1 Design Experiment 1

The purpose of Experiment 1 was to determine whether mice observe the behavior of other individuals based on their learning experience [1]. Thirty-two C57BL/N male mice were prepared and randomly assigned to the Learning and Unlearning groups (Fig. 1). The Learning group was trained in reaching behavior in the reaching room, and the Unlearning group was habituated in the observation room. After the training, the main data collection was conducted in test sessions.

In the test session, we categorized the mice in the observation room and reaching room as cage mates or non-cage mates (Fig. 1; Table 1), and the occurrence of the three categories of behavior, “face to face,” “ambiguous,” and “not paying attention,” was calculated (Fig. 2 and Fig. 3). In summary, Experiment 1 was conducted using a two-factor between-subjects design (2 [learning/unlearning] × 2 [cage mate/non-cage mate]).

We measured the time required by a mouse to complete a single spin before performing the reaching action in the test session to assess social perception. In animals, it is known that trained behavior is facilitated by social perception [1], [2]. Then, we compared the speed of spin in reaching individuals for the following conditions: absence of observers (session 7), presence of an unlearning observer, or presence of a learning observer (Table 3).

The time mice spent close to the slit in the observation room during the test session was then calculated from the video captured by the upper camera to determine if there was a difference in rate of the observer position between the Learning and Unlearning groups in the total time in the test session (Fig. 4; Table 6).

### 2.2 Design Experiment 2

The purpose of Experiment 2 was to evaluate which of the following factors influence the motivation of mice to observe the behavior of other individuals in Experiment 1: the movement intention of other individuals or the situation of the presence of other individuals, and the food table. Thirty male C57BL/N mice were prepared and randomly assigned to the Learning, Unlearning, and Empty groups, with ten animals in each group (Table 8). After three groups were trained to reach for food in the same way, the situations assigned to them in the test session were different as follows. The Learning group observed the reaching behavior of other individuals. The Unlearning group was placed in a situation where unlearned individuals were present in the training room. The Empty group was placed as the situation where the training room was empty.

Twenty animals were prepared for the demonstration of learned reaching or unlearned reaching in the reaching room in the test session (Table 9).

Forty animals (30 in three experimental groups; ten demonstrators for the test session) were trained in reaching behavior. Ten demonstrators of unlearned reaching were habituated as done in Experiment 1. After the training, the main data collection was conducted in a test session.

In the test sessions, the time mice spent close to the slit in the observation room was measured using the videos from the upper camera, and the rate of the observer position being close to the slit for each total time in the test was determined. We compared the values obtained in the three conditions, learning, unlearning, and empty (Table 10). Thus, Experiment 2 had a one-factor, three-level (learning, unlearning, and empty), between-subjects design. We also categorized the occurrence of the three categories (face to face, ambiguous, and not paying attention) in the learning-learning pair, as in Experiment 1 (Fig. 2 and Fig. 3).

### 2.3 Materials

#### 2.3.1 Animals in Experiments 1 and 2

For Experiment 1, 32 male C57BL/N mice (CREA Japan Inc.), with a mean weight of 21.25 g (SD: 1.12 g) and an age of 6 weeks at the time of purchase, were separated into groups of four mice and housed in a temperature-controlled (approximately 23 °C) animal room under a 12 h light/dark cycle (light from 8:00 AM to 20:00 PM) (Table 1). Before the experiments, the mice were provided with food (CE-2, CREA Japan, Inc.) and tap water ad libitum as preliminary breeding for one week. During the behavioral experiments, approximately 1 g of food per day was provided to each mouse after the day's experiment. Tap water was continuously available in their home cages.

For Experiment 2, 50 male C57BL/N mice (CREA Japan Inc.), with a mean weight of 19.25 g (SD: 1.39 g) and an age of 5–7 weeks at the time of purchase, were separated into groups of two or four mice (Table 8 and Table 9). We randomly assigned ten animals to the Learning group, ten to the Unlearning group, and ten to the Empty group. They were all trained in reaching. In addition, twenty animals were prepared as demonstrators of learned reaching or unlearned reaching in the reaching room for the test session. The demonstrators for the unlearned reaching group only exhibited the unlearned reaching behavior in the test session. Before the experiments, the treatment during preliminary breeding was the same as that in Experiment 1, except that the duration was 1–14 days. During the experiment, the feeding and water restriction schedule was the same as that in Experiment 1.

#### 2.3.2 Apparatus in Experiments 1 and 2

The apparatus included a reaching room and an observation room [1]. Both compartments were 10 cm deep, 19 cm wide, and 20 cm tall and were made of transparent acrylic with a feeding table between the two sides. In the reaching room, a slit (10 mm) was created near the feeding table to allow the mice to reach for and grasp a piece of pasta. In the observation room, a slit (1 mm) was created towards the feeding table. The design of the reaching room was according to that described by Farr and Wishaw (2002) [3]. Using a stick that could hold pasta, the experimenter would bring the pasta in front of the slits. We placed two video cameras, one above and another in front of the apparatus, and recorded the animals’ behaviors (60 fps).

### 2.4 Methods

#### 2.4.1 Training in Experiments 1 and 2

The Learning group was trained in reaching behavior twice daily in the reaching room. Reaching behavior was defined as reaching for a food reward (pasta), grasping, and eating it [4]. In a session, twenty rewards (20 trials) were provided to a mouse to allow it to accurately perform the act of reaching for and grasping the pasta; there was a time limit of 20 min for each session. Approximately 1−2 days before the first training, the mice were given pasta and habituated with it. The pasta was cut into a length of approximately 2–3 mm, and each piece weighed 10 mg. The inter-trial interval in a session depended on individual mouse behavior.

In sessions 1 and 2, the mice were trained to reach for and grasp the pasta with their forepaws. From session 3 or 4, the experimenter did not present pasta when the mouse was sitting in front of the slit but presented it when the mouse was positioned away from the slit. This caused the mouse to turn in its spot before reaching for the pasta. Therefore, the mouse does not perform the reaching movement in the absence of the trigger but does so in its presence. Additionally, the movement before reaching could be standardized. In sessions 5−7, we trained the mice to reach for and grasp the pasta only after they had performed the spinning movement.

The mice from the Unlearning group in Experiment 1 and demonstrators for the Unlearning group in Experiment 2 were placed in the observation room for 10 min without pasta while keeping the reaching room empty during each session.

A reaching success rate of over 60% was the criterion for completion of learning. Mice that exceeded this criterion were then subjected to test sessions.

#### 2.4.2 Test session in Experiment 1

Following session 7 of Experiment 1, we immediately conducted an observation test; in this, we determined if mice in the observation room paid attention to the other mice performing reaching behavior and compared the amount of time mice spent close to the slit the reaching room between the Learning and Unlearning groups (Tables 4 and 5). The next day of the test session, the pairs were changed. For Experiment 1, 16 cage mate and 16 non-cage mate pairs of observers and reaching mice were created. Furthermore, the reaching mice were observed once by both the Learning and the Unlearning groups. The combinations of the pairs are shown in Fig. 1 and Table 1.

For the observation test, we manually classified the behavior of the observer during reaching situations into three different categories using a previous study as a reference [1, 2]: “face to face,” “ambiguous,” and “not paying attention” (Fig. 2 and Fig. 3).

We measured the time required to complete a single spin before performing the reaching action in session 7 for the Learning group and the Unlearning group (Table 3). The starting point of the spin was the first frame in the video in which the mouse started spinning after sitting in front of the slit, and the end point was the frame before the one in which the front paws of the mice were away from the ground. We measured the time with a stopwatch.

The time spent close to the slit by the mice in the observation room during the test session was then calculated from the videos of the upper camera using the stopwatch (Fig. 4; Table 6). The observation room was divided into two regions, one on the side of the slit and the other on the opposite side of the slit, to determine if there was a difference in the time spent by the observing individuals in each region. The rate of the observer position was close to the slit was calculated by dividing the time spent near the slit by the total time of the experiment.

#### 2.4.3 Test session in Experiment 2

Following session 7 of Experiment 2, an observation test was conducted, which was similar to that in Experiment 1, except for the conditions. We examined whether the mice that have learned reaching behavior in the observation room spent time close to the slit under three conditions as follows: learning or unlearning mice in the reaching room or empty reaching room (Table 10). We calculated the occurrence rate of the observer's position being close to the slit based on the total time of the test session.

In addition, we measured the time of a single spin in only the learning-learning pair (Table 12) and manually classified the reaching situations into three categories; Face to face, ambiguous, and Not paying attention (Table 13), as in Experiment 1.

## Ethics Statement

All experiments involving animals were performed following the guidelines for animal research at Tohoku University. The experimental protocols were approved by the Ethical Review Board for Animal Study of the Tohoku University School of Medicine (No. 2017–334).

## Declaration of Competing Interest

The authors declare that they have no known competing financial interests or personal relationships that have, or could be perceived to have, influenced the work reported in this article.

## Appendix Supplementary materials

Image, application 1
